# Successful Salvage of Delayed Venous Congestion After DIEP Flap Breast Reconstruction

**Published:** 2019-12-03

**Authors:** Kristopher Katira, Samita Goyal, Chelsea Venditto, John A. LoGiudice, Erin L. Doren

**Affiliations:** ^a^Department of Plastic Surgery, Ochsner Health System, New Orleans, La; ^b^Department of Plastic Surgery, Medical College of Wisconsin, Milwaukee

**Keywords:** DIEP flap, microvascular flap compromise, microsurgery, venous congestion, breast reconstruction

## Abstract

**Objective:** Failure rates of microvascular autologous breast reconstruction are reportedly low. When failure of the microvascular anastomoses does occur, it is most likely to be salvaged if detected early. Flap compromise or venous congestion occurring several weeks later is uncommon and with significantly lower salvage rates. **Methods:** We present a unique case of delayed venous congestion of a single-perforator deep inferior epigastric perforator flap breast reconstruction in which the usual pedicle thrombosis was not identified. Presentation of the flap compromise occurred 72 hours postoperatively and again in the delayed setting 5 weeks after surgery, from suspected compression at the perforator level. **Results:** The deep inferior epigastric perforator flap was successfully salvaged with conservative measures, and the flap healed without fat necrosis or further complication. **Conclusion:** This case highlights the higher risk of flap compromise with reconstructions in a radiated field and potentially with single-perforator flaps.

The benefits of autologous breast reconstruction are well described and supported by patient-reported outcomes data.^1^^-^4 Although flap failure rates are typically cited as 1% to 2%, there are few scenarios among reconstructive breast surgeons that are as devastating to patients and the health care team as a failed reconstruction.^5^^-^7 Many strategies can be employed for flap salvage, depending on the timing and nature of the compromised flap. Anastomotic revision, super-charging, selection of new recipient vessels, and thrombolysis have all been proposed depending on the clinical scenario.^8^

Complications of flap compromise range from partial to complete flap loss and fat necrosis. Fat necrosis rates have been reported to be higher in single- compared with multiple-perforator abdominal flaps in some case series,^9^^,^^10^ while other studies directly contradict this finding.^11^^-^13 Interpreting salvage rates and correlating surgical technique with complications are difficult, given the heterogeneity of flap compromise scenarios, but one clear consensus is that chance of salvage decreases in the delayed setting.^14^^,^^15^

In this report, we discuss the management and salvage of a patient with a single-perforator deep inferior epigastric perforator (DIEP) flap breast reconstruction who presented with delayed venous congestion on postoperative day 3 and again 5 weeks after autologous reconstruction. This case highlights the higher risk of flap compromise in a radiated field and potentially with single-perforator flaps.

## CASE REPORT

**Figure 1:**
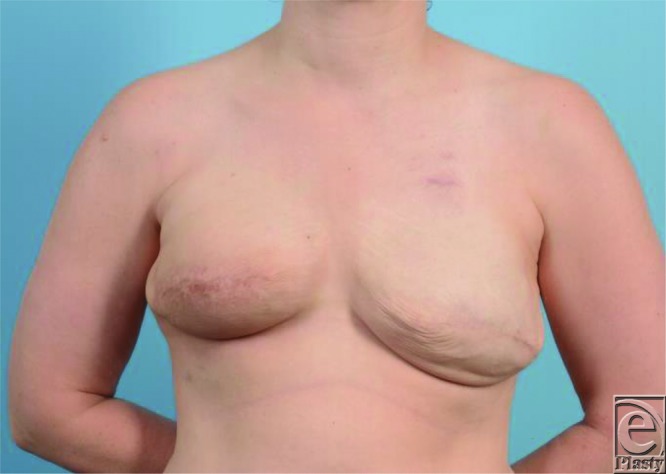
Preoperative photograph. Bilateral tissue expander reconstruction 9 months after completion of right breast radiation therapy.

A 40-year-old woman with stage IIb right invasive ductal carcinoma underwent bilateral skin-sparing mastectomy and immediate subpectoral tissue expander breast reconstruction in anticipation of right breast adjuvant radiation (Fig 1). Nine months following completion of radiation therapy, she elected to undergo bilateral DIEP flap breast reconstruction. She had a body mass index of 25 and no other significant medical history or contraindications to surgery. Computed tomography-angiography of the abdomen showed acceptable perforators for reconstruction.

Her DIEP reconstruction was uneventful. Single-perforator flaps were raised in both hemiabdomens on large periumbilical perforators. The abdominal flaps were transferred to the contralateral chest with a 90° rotation. The left hemiabdominal flap perforator was located 4.8 cm to the left of the umbilicus and 2.3 cm caudal. The right hemiabdominal flap perforator was located 5.3 cm to the right of the umbilicus and 0.6 cm caudal. Clinical and indocyanine green laser angiographic examinations of the fully dissected abdominal flaps showed excellent perfusion. On each side, a single venous anastomosis was performed with 3.0-mm coupler between the larger medial vena comitans and the anterograde internal mammary vein at the level of the third rib. Flaps were placed in the prepectoral plane. Lower pole was heavily radiated, and poorly expanded mastectomy skin was excised. An identical procedure was performed on the contralateral nonradiated breast for symmetry.

**Figure 2:**
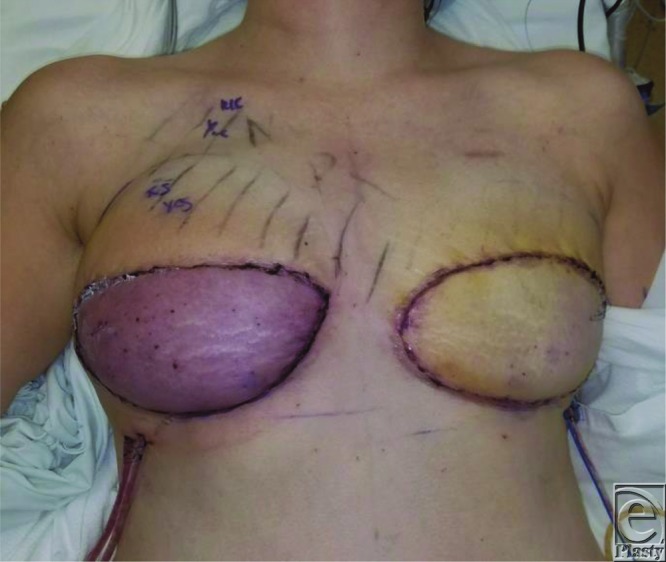
Postoperative day 4 acute venous congestion of the right deep inferior epigastric perforator flap.

Cutaneous Doppler flap checks proceeded postoperatively, with noted strong arterial and venous signals. On postoperative day 3, the patient got out of the shower and was noted to have acute venous congestion of the right flap. A venous signal at this point was still noted and the congestion resolved by the time the operative team was assembled 1 hour later. Upon ambulation postoperative day 4, she had recurrent venous congestion and loss of venous signal and was urgently taken to the operating room for exploration (Fig 2).

Exploration of the flap proceeded as follows: Upon releasing her superior flap incision, the congestion resolved and a venous signal returned. The vascular anastomosis was inspected for thrombosis, kinking, twisting, tension, compression from hematoma, all of which were excluded. Continued monitoring of the flap intraoperatively did reveal that the venous signal was lost when the superior mastectomy skin was reapproximated to the flap skin paddle.

**Figure 3:**
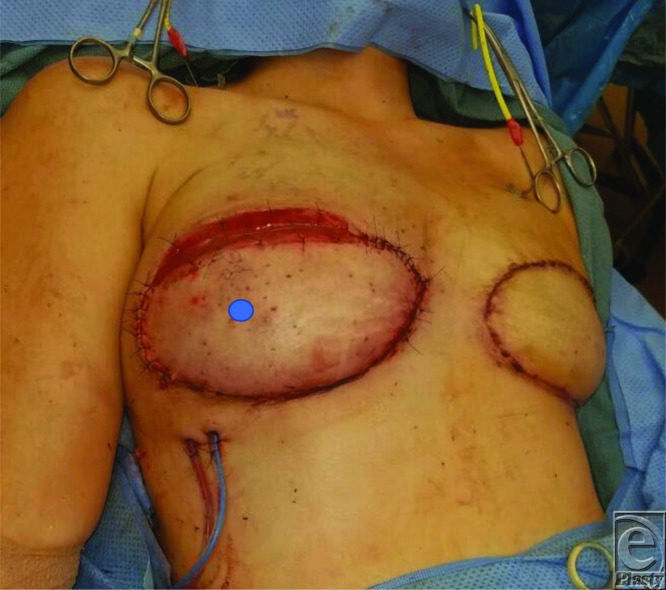
Intraoperative photograph of the right radiated mastectomy skin flap released from the deep inferior epigastric perforator flap skin paddle and resolution of venous congestion. Bilaminar wound matrix interposed between the flap and mastectomy skin. (Blue circle marks approximate the perforator location.)

Observation revealed that the perforator may have been kinking between the pectoralis muscle and the remnant abdominal fascial cuff around the perforator. Despite making a full-thickness myotomy in the pectoralis muscle under the perforator, the venous signal was again lost when the mastectomy skin was opposed to the flap. The flap inset was therefore left open, and the cause of congestion was determined to be compression (Fig 3).

**Figure 4:**
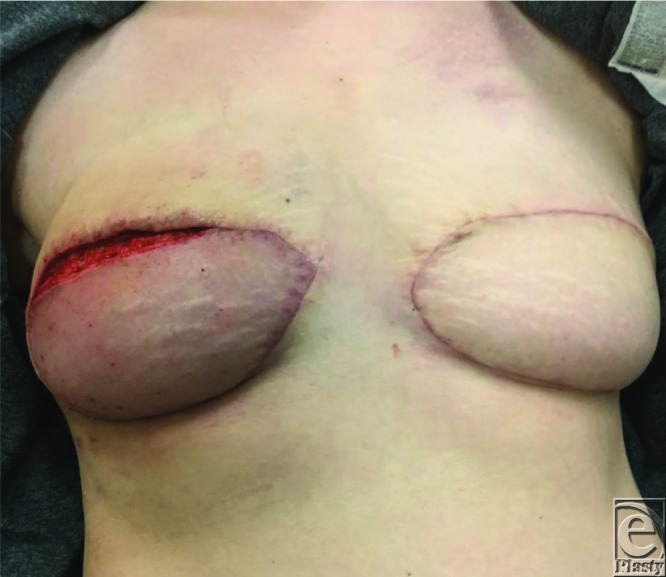
Recurrent venous congestion of the right deep inferior epigastric perforator flap noted 5 weeks postoperatively.

**Figure 5:**
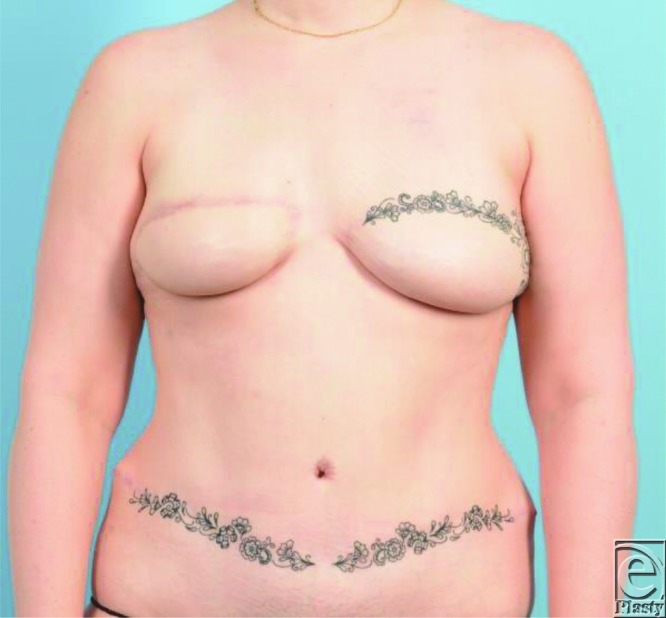
Final result over 1 year later.

The flap completely recovered and had a normal examination until 5 weeks postoperatively when the patient presented again with acute venous congestion (Fig 4). Her superior mastectomy skin had been closed in clinic 2 days prior. The congestion was noted to resolve in the supine position and again upon release of the superior incision. The patient was then monitored in the hospital with leech therapy for 5 days. The flap congestion completely resolved, and the patient had no further complications or fat necrosis of the flap. Her open wound was allowed to heal by secondary intention, and she was offered scar revision but declined. Her final result can be seen in Figure 5 over 1 year later.

## DISCUSSION

In both cases of venous compromise in this patient, positional changes preceded the onset of venous compromise and release of the mastectomy skin from the flap resulted in improvement in the flap circulation. The relative inelasticity of her radiated mastectomy skin, as compared with the contralateral side, clearly contributed to this phenomenon. After a critical period of postoperative edema or with position changes after skin closure, venous occlusion occurred around the abdominal fascial cuff around the perforator, a process that may have been exacerbated by positional change.

This mechanism of venous compromise could have been avoided had we raised a flap on multiple perforators. Numerous studies have focused on the relative merits of single- versus multiple-perforator dissections, especially as they relate to flap perfusion, complication rates, or the incidence of fat necrosis.^9^^-^11 There is, however, no clear consensus about the relative merits of single- versus multiple-perforator flaps in terms of fat necrosis rates among these studies. Chang et al^5^ reported higher flap loss rates with single-perforator flaps in their series of 2138 breast free flaps. That said, multiple-perforator flaps take longer to raise and can result in more extensive donor site morbidity. In any case, limiting the size of the myofascial cuff around a single perforator could have theoretically prevented venous compromise in our patient with a single-perforator flap, but this also takes additional time and risks injury to the perforator, particularly when it exits at an inscription point.

Radiation-related complications in breast reconstruction are well known and likely contributed to recurrent flap compromise in our patient. Many authors have discussed radiation injury to tissues, in particular as it relates to the appropriate timing of reconstructive surgery after radiation therapy and the incidence of complications when performing reconstruction in a radiated wound bed.^16^^,^^17^ Anecdotally, our own experience performing reconstruction in radiated fields supports these observations. In general, we do not offer reconstruction or revisionary surgery immediately after radiation therapy, preferring to wait 9 months to 1 year.

One clear consensus in the literature is that delayed flap compromise portends a very low risk of flap salvage. Largo et al^14^ reported a series of 10 patients with autologous breast reconstruction presenting with flap compromise after discharge from the hospital, and none of these flaps were successfully salvaged despite reoperation procedures. Of the 47 takebacks in the Mirzabeigi et al^15^ series, none of the flaps were salvaged beyond 96 hours. In the Chen et al^18^ series of 1142 free flaps, the lowest rates of flap salvage were reported for those flaps presenting with vascular compromise 1 or more weeks after the initial free tissue transfer procedure. In our case of successful salvage of a recurrently venous-compromised flap presenting as late as 5 weeks postoperatively, expediency of intervention was paramount. Had we not intervened in a timely fashion, it is not unreasonable to suppose that pedicle thrombosis would have resulted. The importance of skilled inpatient monitoring of free flaps cannot be overstated. In practices where patients travel long distances for surgery or in centers abiding by enhanced recovery pathways aimed at minimizing duration of hospital stays, early detection of flap compromise and expedient operative exploration may be problematic.

Interestingly, salvage of compromised flaps has been reported in the delayed setting without exploration. Yoon and Jones^19^ presented a meta-analysis of a heterogeneous group of compromised flaps in the literature that were not explored, and 32 of 43 unexplored flaps remained viable. On the basis of observations of the timing of flap compromise in this heterogeneous group of head and neck, lower extremity, and breast cases, the authors concluded that some venous-compromised flaps could be salvaged without exploration as early as 4 days postoperatively.^19^ Although it is possible that neovascularization and angiogenesis could eventually support a flap independent of the microanastomoses, the timing of revascularization would likely depend on a number of factors, including the type of reconstruction and the vascularity/radiation status of the wound bed. While we would offer exploration to any patient in the acute postoperative period, operative management decisions in the delayed setting are less clear, especially in light of these findings. Regardless of the timing of presentation, suture release or partial de-inset of the flap should be considered as part of the initial management of any venous-compromised flap.^8^

In conclusion, we present a unique case of intermittent and delayed venous congestion of a single-perforator microvascular DIEP flap. The cause of the congestion was determined to be compression at the perforator level in a previously radiated breast, exacerbated by positional changes. This case highlights the importance of performing reconstruction carefully in a radiated field and suggests the use of multiple perforators or near skeletonization of the single perforator to avoid similar complications in the future. Close monitoring and expedient exploration are useful in preventing progression to thrombosis in cases of compromised flaps.
